# SFRP5 Enhances Wnt5a Induced-Inflammation in Rheumatoid Arthritis Fibroblast-Like Synoviocytes

**DOI:** 10.3389/fimmu.2021.663683

**Published:** 2021-06-15

**Authors:** Dorra Elhaj Mahmoud, Wajih Kaabachi, Nadia Sassi, Amel Mokhtar, Myriam Moalla, Lobna Ben Ammar, Samia Jemmali, Sonia Rekik, Lamjed Tarhouni, Maryam Kallel-Sellami, Elhem Cheour, Lilia Laadhar

**Affiliations:** ^1^ Immuno-Rheumatology Research Laboratory, Rheumatology Department, La Rabta Hospital, University of Tunis-El Manar, Tunis, Tunisia; ^2^ Medicine School of Tunis, University of Tunis-El Manar, Tunis, Tunisia; ^3^ Rheumatology Department, La Rabta Hospital, Tunis, Tunisia; ^4^ Department of Hand and Reconstructive Surgery, Kassab Institute of Traumatic and Orthopedic Surgery, Tunis, Tunisia

**Keywords:** rheumatoid arthritis, Wnt pathway, FLS, fibrocytes, inflammation

## Abstract

**Background:**

Tissue derived fibroblast-like synoviocytes (td-FLS) are key actors in pannus formation and contribute to joint destruction and inflammation during rheumatoid arthritis (RA). Several members of the Wnt family, including Wnt5a, may contribute to RA td-FLS activation and can potentially serve as therapeutic targets.

**Objective:**

The present work aimed to investigate the expression of Wnt5a signaling elements in RA td-FLS and their potential precursors (fluid derived (fd) FLS and fibrocytes). We also studied the role of Wnt5a in RA td-FLS pro-inflammatory activity and whether the inhibitor SFRP5 could restore Wnt5a-induced synovial dysfunction *in vitro*.

**Materials and Methods:**

The levels of Wnt5a, SFRP5, Wnt5a receptors/coreceptors and Wnt5a pro-inflammatory targets were determined in cultured RA td-FLS, fd-FLS and fibrocytes using qPCR under basal conditions. The expression of pro-inflammatory molecules was assessed after RA td-FLS stimulation with Wnt5a and SFRP5 at different time points.

**Results:**

Our data showed that td-FLS, fd-FLS and fibrocytes from patients with RA expressed similar levels of Wnt5a and a set of Wnt5a receptors/coreceptors. We also demonstrated that Wnt5a stimulated the expression of the pro-inflammatory targets, especially IL1β, IL8 and IL6 in RA td-FLS. Wnt5a-induced inflammation was enhanced in the presence of SFRP5. Furthermore, Wnt5a alone and in conjunction with SFRP5 inhibited the gene expression of TCF4 and the protein levels of the canonical coreceptor LRP5.

**Conclusion:**

Wnt5a pro-inflammatory effect is not inhibited but enhanced by SFRP5 in RA td-FLS. This research highlights the importance of carefully evaluating changes in Wnt5a response in the presence of SFRP5.

## Introduction

Rheumatoid arthritis (RA) is a systemic autoimmune disease that affects approximately 1% of population worldwide (1). Synovial lining fibroblast-like synoviocytes (also called tissue derived fibroblast-like synoviocytes (td-FLS)) are mesenchymal cells that display many characteristics of fibroblasts including expression of type I and V collagens, fibronectin vimentin, and CD90. During RA, these cells exhibit multipotent pathogenic proprieties (2, 3). Explanations for RA td-FLS aggressiveness include migration of new cells from the blood and bone marrow into the inflamed joint (4).

It is also possible that circulating fibrocytes can migrate into the inflamed joint and then differentiate along a fibroblast-like synoviocyte pathway. Furthermore, synovial fluid (SF) from RA patients has been described to give rise to fluid derived FLS (fd-FLS), which are morphologically similar to td-FLS (5). These cells are also described in osteoarthritis (OA) synovial fluid and exhibit similar pro-inflammatory characteristics to OA td-FLS (6).

The activation of td- FLS during RA results from many alterations in several homeostatic mechanisms and signals including the Wnt pathway. This signaling is involved in many biological processes including embryonic development, cell migration, tissue organization and human diseases (7, 8). Wnt5a, a prototype Wnt, is involved in several inflammatory diseases. It mediates the production of many inflammatory molecules including IL1β, IL6 and IL15 during autoimmune disorders (4, 9). In RA td-FLS, Wnt5a expression is enhanced (4, 9). The signaling pathways induced by Wnt5a are mediated by canonical (β-catenin-dependent) or noncanonical (β-catenin independent) pathways (10). The β-catenin dependent signaling pathway is by far the best-characterized and comprises signal transduction through the accumulation of β-catenin protein as well as specific T-cell factor/lymphoid enhancer factor (LEF/TCF) family transcription factors (11, 12)

Wnt5a binds several receptors, including members of frizzled (Fzd) receptor family, Ror2 and Ryk (9, 13). In addition to Fzd receptors, the canonical Wnt pathway requires transmembrane proteins that belong to a subfamily of low-density lipoprotein (LDL) receptor- related proteins (LRP) such as LRP5 (9, 13). The interaction of Wnt5a with Fzd receptors is antagonized by secreted frizzled -related proteins (SFRP) such as SFRP5. However, a previous study postulated that SFRP could also promote long-term Wnt signaling in certain instances by binding to Wnt proteins and protecting them from degradation (14). Data on the relationship between Wnt5a and SFRP5 in humans are limited and inconsistent (15, 16). Furthermore, the Wnt5a ligand can have disparate effects on cells depending on receptor availability. Therefore, the cellular context dictates the effect of Wnt5a (17). Even though td-FLS perform key functions in RA pathogenesis and joint inflammation, the expression of Wnt5a signaling elements in these cells and their previously described precursors (fd-FLS and fibrocytes) has not been well studied.

We investigated whether the three cell types express Wnt5a, SFRP5, Wnt5a receptors/coreceptors and Wnt5a principal targets. We also studied the involvement of Wnt5a in td-FLS pro-inflammatory activities during RA.

## Materials and Methods

### Patients and Samples

The study included 14 patients with RA. Each patient donated a unique biological sample (peripheral blood, synovial fluid, or synovial tissue). Peripheral blood samples were obtained from five patients with RA. Synovial tissues were obtained from five patients with RA during total joint replacement or synovectomy. Synovial fluid (SF) samples were collected from the knees of four patients with active RA using a standard sterile procedure. The median age of all patients was 57 years old (range 35–79) with a disease duration of 15 years (7–26). RA patients fulfilled the criteria for the ACR/EULAR 2010 (18). RA patient characteristics are detailed in **Table 1**. As a control, 3 synovial membranes from patients with osteoarthritis (OA) were used. These tissues were collected at the time of total knee replacement surgery. The experimental protocols used in this study were approved by the ethics committee of la Rabta hospital, Tunis, Tunisia. Informed consent was obtained from each patient included in this study.

**Table 1:** RA Patient characteristics.

**Donation**	**Blood (n = 5)**	**Synovial fluid (n = 4)**	**Synovial Tissue (n = 5)**
**Age, years^a^**	59 (50-79)	60 (52-79)	50 (35-64)
**Disease duration, years^a^**	14 (7-26)	18 (12-26)	14 (8-20)
**Female sex, n (%)**	3/5	2/5	3/5
**Site**	Peripheral blood	Knee	Wrist (1 synovectomy and 4 total joint replacement)
**Volume (ml)**	10-12	1-20	
**Sample collection**	Sterile heparinized tubes	sterile syringe	50 ml Falcon centrifuge tube with medium
**ACPA^b^ + and RF^c^+, n (%)**	5/5	4/4	N/A
**CRP^a^ mg/l**	42.9 (5-164.8)	16.4 (7.6-13.6)	N/A
**Medications, n**	5/5	4/4	N/A
**TNF-inhibitors^d^, n**	0	0	
**Methotrexate, n**	5/5	2/4	
**Prednisone, n**	0	2/4	

^a^Median and range, ^b^Anti-citrullinated Protein Antibody, ^c^Rheumatoid Factor, ^d^TNF inhibitors = Humira, Enbrel, Simponi, Remicade, N/A data at the time of donation is not available.

### Td-FLS Isolation and Culture

RA and OA synovial fragments were washed with sterile phosphate-buffered saline (PBS). The tissues were minced and treated overnight with 1.5 mg/ml of collagenase A (Roche^®^) in Dulbecco’s modified Eagle medium (DMEM) (Gibco^®^) at 37°C. Dissociated cells were then centrifuged at 3500 rpm for 3 min, washed and resuspended in DMEM supplemented with 20% fetal bovine serum (FBS) and 1% penicillin- streptomycin (Gibco^®^). The cell pellet was seeded in T-75 culture flasks (Nunc^®^) and cultured at 37°C with 5% CO2. After 72h, non-adherent cells were removed, and adherent cells were kept at 37°C in 5% CO2. The medium was replaced every 3 days. After 10-14 days of culture, adherent cells were trypsinized using trypsin-EDTA (Gibco^®^) and transferred to new flasks with a density of 5.10^5^ cells/flask. Passage 3 cells were morphologically homogenous and were used for experiments.

### Fd-FLS Isolation and Culture

RA synovial Fluid was centrifuged at 1000 rpm for 30 min and cell pellets were resuspended in DMEM containing 20% FBS and 1% penicillin-streptomycin in a T-75 flask. After 72h, non-adherent cells were removed by replacing the culture medium with a fresh one, and attached cells were cultured in the above -described culture medium. Passage 3 cells were used for RNA and protein extraction.

### Fibrocyte Isolation and Culture

RA Fibrocytes were generated in peripheral blood mononuclear cell (PBMC) cultures. Peripheral blood was mixed with DMEM (2:1), layered over Ficoll/Plaque (Sigma^®^) and centrifuged at 1800 rpm for 30 min. PBMC were collected, washed then cultured in DMEM supplemented with 20% FBS and 1% penicillin-streptomycin (Gibco^®^). On the fifth day, DMEM was replaced with a fresh medium. After 14 days, the adherent fibrocytes were trypsinized and transferred in 6 well plates (Nunc^®^). The viability of the isolated cells was assessed by the trypan blue exclusion method. Fibrocytes were identified based on the previously described criteria: adherent cells with elongated spindle-shaped morphology distinct from lymphocytes or adherent monocytes and oval nuclei (19, 20). Fibrocytes were used on day 17 for RNA and protein extraction.

### RA td-FLS Stimulation

RA td-FLS were seeded in 6-well plates at a density of 12 × 10^4^ cells/well and maintained at 5%CO_2_ for 24h. Cells were treated with recombinant Wnt5a (300 ng/ml) (R&D systems^®^) and/or recombinant SFRP5 (R&D systems^®^) for 4h and 24 h. For the combined Wnt5a/SFRP5 treatment, SFRP5 was added 10 min before Wnt5a stimulation. Td-FLS were collected and used for RNA and/or protein extraction.

### RNA Isolation and cDNA Synthesis

Total RNA was extracted using the “PureLink^®^ RNA Mini Kit (Invitrogen^®^) according to the manufacturer’s protocol. Sample concentrations were measured (Nanodrop 2000^®^). Reverse transcription was realized as follows: 1μl of random primers (Invitrogen^®^) was added to the RNA solution and incubated 10 min at 70°C followed by 5 min incubation on ice. dNTP, M-MLV, RNase out, 5X buffer and DTT (Invitrogen^®^) were added to RNA solution, incubated for one hour at 37°C then 10 min at 70°C.

### Quantitative PCR

Real-time PCR was performed using ABI7500^®^ qPCR System using the SYBR Green qPCR Kit (Invitrogen^®^) with the primers listed in **Table 2**. A reaction volume of 20 μl (2.0 μl cDNA) was amplified for 40 cycles after initial denaturation (95°C, 10 min) with the following parameters: 95°C for 15 s, 60°C for 1min. Specificity was checked and reaction concentration was optimized before sample analysis. Samples were run in duplicate or triplicate, and a relative quantification of mRNA level was performed using glyceraldehyde-3-phosphate dehydrogenase (GAPDH) as an endogenous reference. The basal gene expression between RA td-FLS, RA fd-FLS and fibrocytes were analyzed using the ΔCT method; all other data (stimulation experiments) were presented as fold change using the 2^-ΔΔ^CT method.

**Table 2:** Primers (Invitrogen^®^) used for qPCR.

**Targets**	**Forward**	**Reverse**
**Wnt5a**	CAACTGGCAGGACTTTCTCA	TTCTTTGATGCCTGTCTTCG
**SFRP5**	CACAAGTTCCCCCTGGACAA	TGTGCTCCATCTCACACTGG
**Fzd4**	TACCTCACAAAACCCCCATCC	GGCTGTATAAGCCAGCATCAT
**Fzd5**	GTCACACCCGCTCTACAACA	CACTGAAGGACGGCTGGTAG
**Ror2**	ATGGAACTGTGTGACGTACCC	GCGAGGCCATCAGCTG
**Ryk**	TCTACCTGAGCGAGGACGAG	CCACTTGGAATCCCAGCTTA
**LRP5**	ATGGGCGCCAGAACATCAA	AGATGTCGATGCTGAGGTCGTG
**TCF4**	CTGCCTTAGGGACGGACAAAG	TGCCAAAGAAGTTGGTCCATTTT
**β-catenin**	TCTGAGGACAAGCCACAAGATTACA	TGGGCACCAATATCAAGTCCAA
**IL6**	GTAGCCGCCCCACACAGA	CATGTCTCCTTTCTCAGGGCTG
**IL8**	ATAAAGACATACTCCAAACCTTTCCAC	AAGCTTTACAATAATTTCTGTGTTGGC
**IL1β**	AAATACCTGTGGCCTTGGGC	TTTGGGATCTACACTCTCCAGCT
**CCL2**	CAGCCAGATGCAATCAATGCC	TGGAATCCTGAACCCACTTCT
**CXCL10**	GAGCCTACAGCAGAGGAACC	GAGTCAGAAAGATAAGGCAGC
**COX2**	CCCATGTCAAAACCGAGGTG	CCGGTGTTGAGCAGTTTTCTC
**GAPDH**	ACTTCAACAGCGACACCCACTC	TACCAGGAAATGAGCTTGACAAAG

### Western Blotting

Total protein extraction was realized using the “Qproteome mammalian protein prep kit” (Qiagen^®^) according to the manufacturer’s protocol. Protein concentrations were measured and 40µg of proteins were loaded into an 8% sodium dodecyl sulfate-polyacrylamide gel electrophoresis (SDS-PAGE) and transferred into nitrocellulose membranes (Biorad^®^). Membranes were blocked with 5% nonfat dry milk in PBS-0.5% Tween 20 (PBS-T) at room temperature for 1h, followed by overnight incubation at 4°C with primary antibodies against alpha-1 type I collagen (ColIA1), fibronectin, LRP5, GAPDH or β-actin (R&D systems^®^). The membranes were washed 3 times with PBS-T and incubated 1h at room temperature with horseradish peroxidase-conjugated secondary antibodies (RnDsystems^®^). After PBS-T washing, membranes were incubated for 1 min with the enhanced chemiluminescence detection solution (ECL) (Perkin Elmer^®^) and signals were detected using Biomax MR^®^ Films *(*Kodak^®^).

### Statistical Analysis

Since sample size is not large enough to verify the normality of the data, significance was determined with a non-parametric Kruskal Wallis test using Graph Pad Prism 6. If the Kruskal Wallis test rejected the null hypothesis a post-hoc test was run for multiple comparisons using XLSTAT. Significance is represented as follows: *p<0.05 and **p<0.01.

## Results

### Expression of Wnt5a in RA *vs.* OA td-FLS

**Figure 1:**
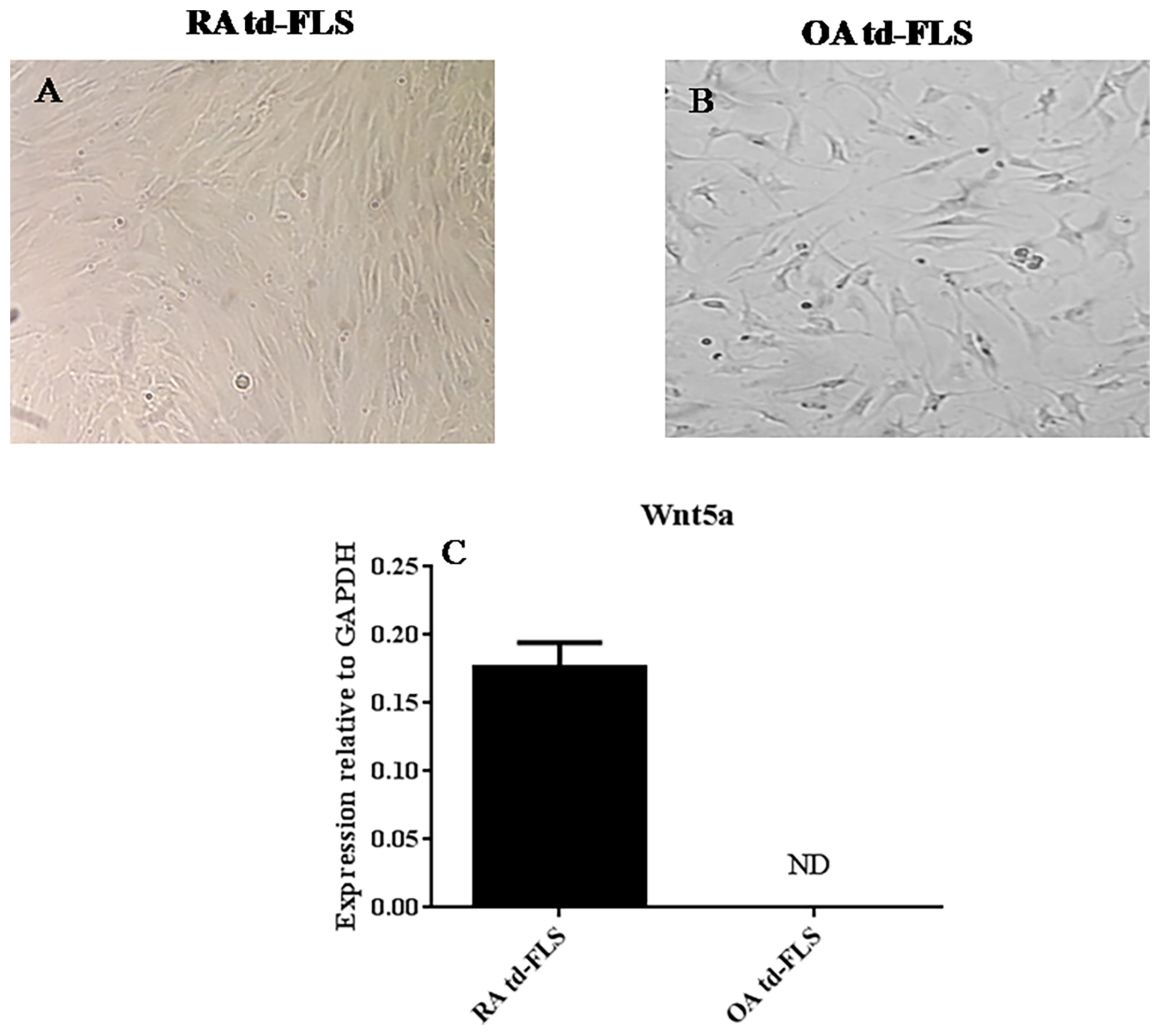
Comparison of Wnt5a expression in td-FLS from patients with RA and OA: Third passage RA td-FLS **(A)**. Third passage OA td-FLS **(B)**. Wnt5a mRNA expression in RA td-FLS (n=3) and OA td-FLS (n=3) **(C)**. Wnt5a was expressed in RA td-FLS but not in OA td-FLS. ND, Not detected. Results are shown as the mean ± SD.

To determine whether Wnt5a expression is specific to RA FLS, we first cultured td-FLS from patients with RA or OA for 3 passages (**Figures 1A, B**). Then, we analyzed the basal mRNA expression of Wnt5a. The real-time PCR showed that Wnt5a was expressed in RA td-FLS but not in OA td-FLS (**Figure 1C**).

### Phenotypic Identification of RA td-FLS Eventual Precursors: RA fd-FLS and RA Fibrocytes

**Figure 2:**
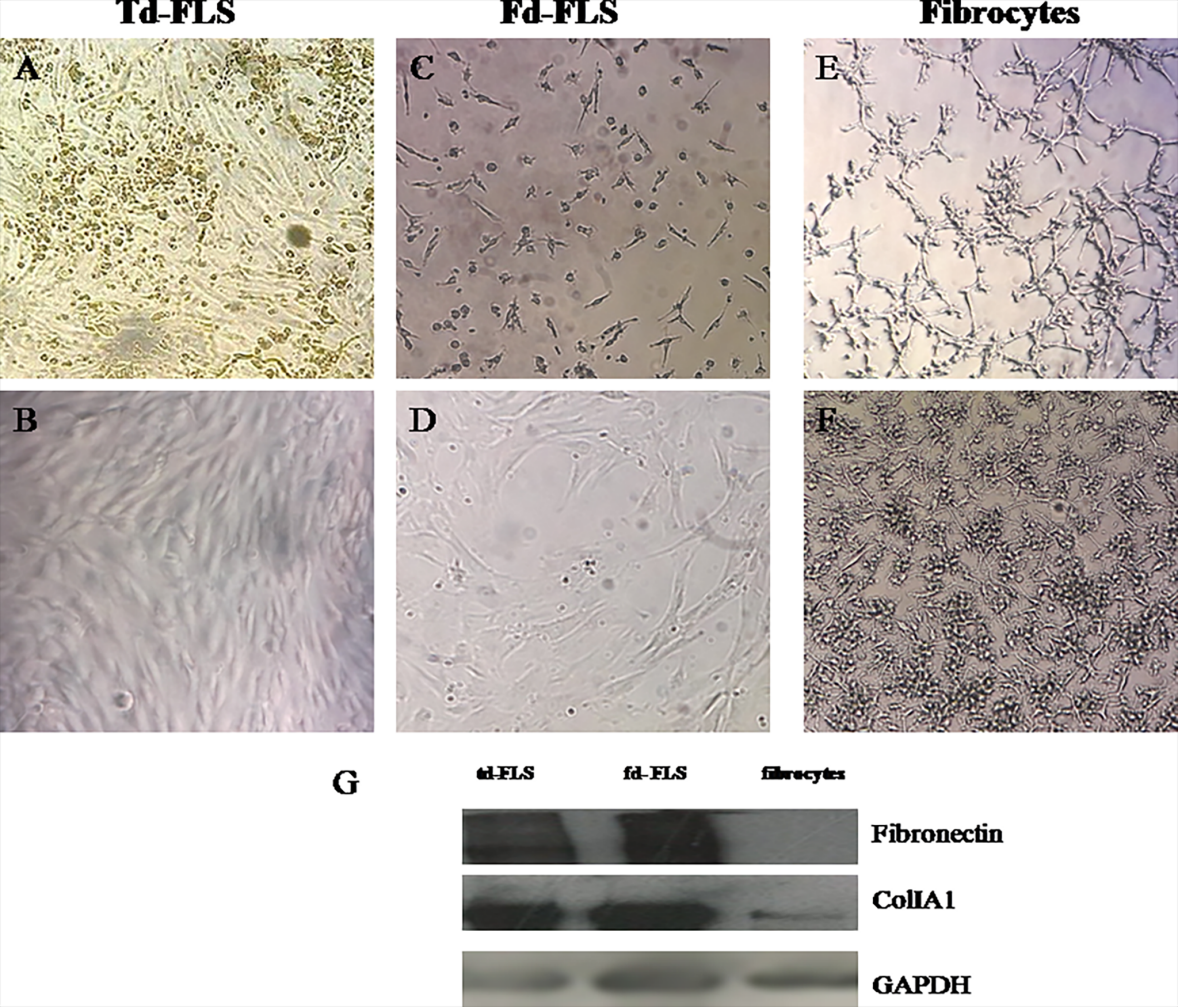
Light microscopic features and mesenchymal marker expression in td-FLS, fd-FLS and fibrocytes from patients with RA: Spindle-shaped adherent cells started to appear in synovial fluid cultures from patients with RA after few days of culture **(C)**. Third passage fd-FLS showed a spindle-shaped appearance **(D)** similar to RA td-FLS **(A, B)**. Fibrocytes from PBMC of patients with RA displayed some features of td-FLS, such as a fibroblast-like morphology. Fibrocytes showed cytoplasmic projections and a slim bipolar-shape **(E, F)** (original magnification ×100). Western blot analysis showed fibronectin, ColIA1 and GAPDH expression in fd-FLS, td-FLS and fibrocytes from patients with RA **(G)**. The Western blots are representative of one experiment out of two.

As for td-FLS, successful fd-FLS and fibrocyte cultures were obtained from synovial fluids and blood of patients with RA. After 3 passages, td-FLS and fd-FLS from patients with RA showed similar morphology with a spindle-shaped appearance and few branched cytoplasmic processes (**Figures 2A–D**). Cultured fibrocytes had a spindle-shaped morphology with numerous cytoplasmic projections (**Figures 2E, F**). Since fibronectin and collagen I (colI) are mesenchymal markers, we evaluated their expression in RA td/fd-FLS from passage 3 and RA fibrocytes after 17 days of cultures using Western blot. As shown in **Figure 2G**, only RA td-FLS and RA fd-FLS expressed fibronectin. The three cell types expressed ColIA1. The higher levels of ColIA1 were observed in RA FLS (td and fd) compared to fibrocytes.

### Expression of Wnt5a and SFRP5 in RA td-FLS, RA fd-FLS and RA Fibrocytes

After obtaining homogenous cultures, we investigated the expression of Wnt5a and SFRP5 genes in td-FLS, fd-FLS and fibrocytes from patients with RA.

**Figure 3:**
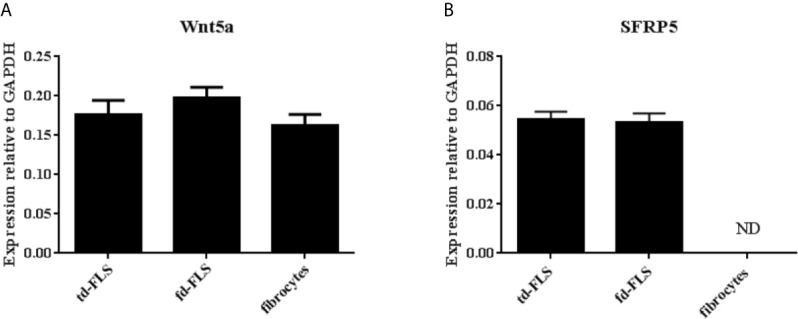
Expression of Wnt5a and SFRP5 in td-FLS, fd-FLS and fibrocytes during RA. Wnt5a gene level profiles in the three cell types by quantitative RT-PCR: No significant differences were found in mRNA levels **(A)**. SFRP5 mRNA expression: SFRP5 was expressed in td-FLS and fd-FLS but not in fibrocytes (n=3-4) **(B)**. ND, Not detected. Results are shown as the mean ± SD.

No significant difference in Wnt5a transcript level was found between the three cell types (**Figure 3A**). SFRP5 mRNA was detected in RA td-FLS and RA fd-FLS but not in RA fibrocytes (**Figure 3B**).

### Expression of Wnt5a Receptors and Coreceptors in RA td-FLS, RA fd-FLS, and RA Fibrocytes

**Figure 4:**
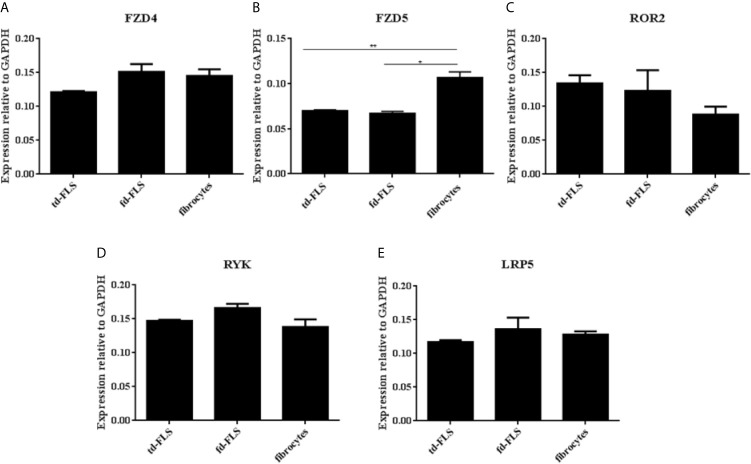
Expression of Wnt5a receptors and coreceptors in td-FLS, fd-FLS and fibrocytes during RA: Relative mRNA levels of Fzd4, Ror2, Ryk and LRP5 in the three cell types: No difference was detected between the cells using qPCR analysis **(A, C–E)**. Fzd5 was highly expressed in fibrocytes compared to td-FLS and fd-FLS (n=3-4) **(B)**. Results are shown as the mean ± SD *Statistically significant difference p < 0.05, **statistically significant difference p < 0.01, using non-parametric Kruskal Wallis test with a *post hoc* test.

To examine whether RA td-FLS, RA fd-FLS and RA fibrocytes express receptors/coreceptors for Wnt5a, we analyzed the mRNA expression for Fzd4, Fzd5, Ror2, Ryk and LRP5 by qPCR. As shown in **Figure 4**, the Fzd5 mRNA level was significantly higher in RA fibrocytes than in td-FLS and fd-FLS. Therefore, no differential expression in Fzd4, Ror2, Ryk and LRP5 level was found between the three cell types.

### Expression of Inflammatory Mediators in RA td-FLS, RA fd-FLS, and RA Fibrocytes

In order to obtain a basic measure of cultured RA td-FLS, fd-FLS and fibrocyte inflammatory profile, we examined the mRNA expression of the inflammatory mediators: IL6, IL1β, IL8, CXCL10, CCL2 and COX2.

**Figure 5:**
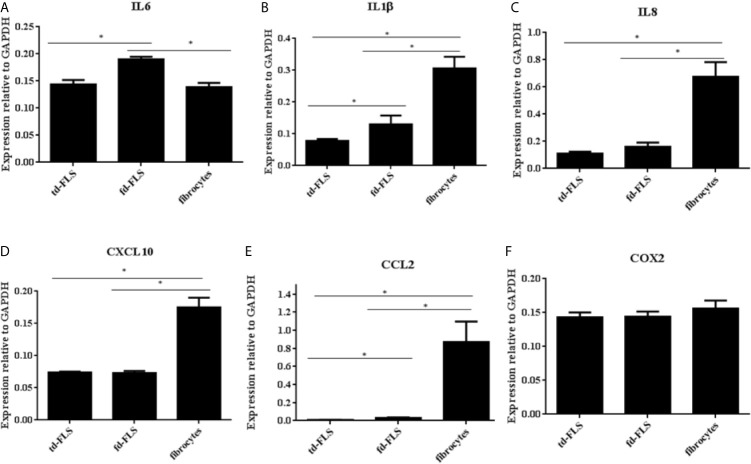
Expression of Wnt5a pro-inflammatory targets in td-FLS, fd-FLS and fibrocytes during RA. IL6 was highly expressed in fd-FLS compared to td-FLS and fibrocytes **(A)**. IL1β and CCL2 were expressed by the three cell types **(B, E)**. The higher mRNA levels for IL1β and CCL2 were found in fibrocytes followed by fd-FLS. IL8 and CXL10 expression profiles were greater in fibrocytes than in td-FLS or fd-FLS **(C, D)**. Relative mRNA levels of COX2 in the three cell types **(F)**: No difference was detected between the cells using qPCR analysis (n=3-4). Results are shown as the mean ± SD.*Statistically significant difference p < 0.05 using non-parametric Kruskal Wallis test with a *post hoc* test.

We found a significantly higher expression of IL6 in RA fd-FLS compared to td-FLS and fibrocytes (**Figure 5A**). The mRNA expression levels of IL1β, IL8, CXCL10 and CCL2 were significantly higher in RA fibrocytes than in td-FLS and fd-FLS (**Figures 5B–E**). Furthermore, IL1β transcript levels were greater in RA fd-FLS when compared to RA td-FLS (**Figure 5B**). No differential COX2 expression levels were found between the three cell types (**Figure 5F**).

### Effect of Wnt5a on Inflammatory Mediator Synthesis by Activated RA td-FLS

The above findings prompted us to explore the involvement of Wnt5a in the pro-inflammatory activity of RA td-FLS. The Wnt5a stimulated expression profile of pro-inflammatory mediators in RA td-FLS was analyzed using qPCR after 4h and 24h of stimulation.

**Figure 6:**
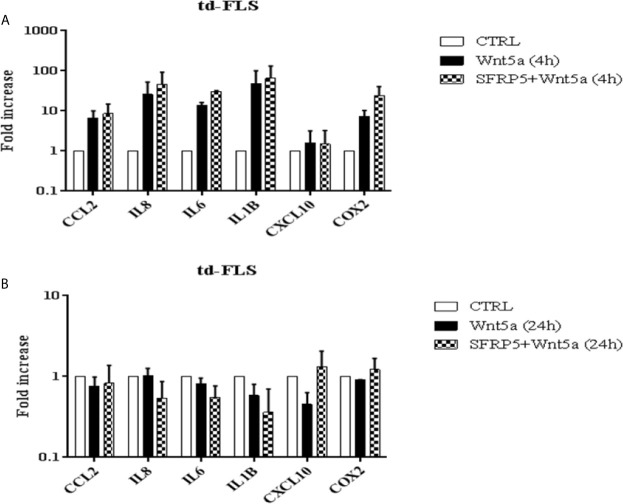
Effect of Wnt5a on the inflammatory response of RA td-FLS. RA td-FLS (passage 3) were stimulated with recombinant Wnt5a (300 ng/ml) in the presence or absence of SFRP5 (1 mg/ml) for 4 h **(A)** and 24h **(B)**. Cytokine expression profiles (CCL2, IL8, IL1β, CXCL10 and COX2) were determined in cell culture using qPCR. Gene expression levels in unstimulated cells were assumed to be 1. Data are means ± SD of two independent experiments each performed in triplicates using at least two different lots of each of the recombinant Wnt proteins.

As shown in **Figure 6A**, IL1β expression was enhanced robustly, more than 48 times over the untreated cells after 4h of treatment. IL8, IL6, CCL2 and COX2 were also rapidly up-regulated (25.7 fold, 13.7 fold, 8.5 and 7.4 fold respectively). CXCL10 was up-regulated mildly (1.5 fold) after 4 h of treatment. We sought if the inflammatory profile induced by Wnt5a in RA td-FLS was inhibited by the Wnt5a soluble regulator: SFRP5. RA td-FLS cytokine production induced by Wnt5a was enhanced in the presence of SFRP5. The combined Wnt5a/SFRP5 treatment induced a massive expression of IL1β, IL8, IL6, COX2 and CCL2 (65.5 fold, 45,2 fold, 30.1 fold, 24.2 fold and 8.5 fold respectively).

At 24h of Wnt5a stimulation, IL1β, IL8, IL6 and CCL2 expression declined and dropped under the initial levels found in the untreated cells (**Figure 6B**).

### Effect of Wnt5a on the Principal Canonical Wnt Component Expression

Finally, we analyzed whether Wnt5a alone or associated with SFRP5 changed the expression of the principal canonical Wnt elements. We analyzed the gene expression levels of β-catenin and TCF4. We also evaluated the protein expression of the coreceptor LRP5 which is crucial for the canonical Wnt activation.

**Figure 7:**
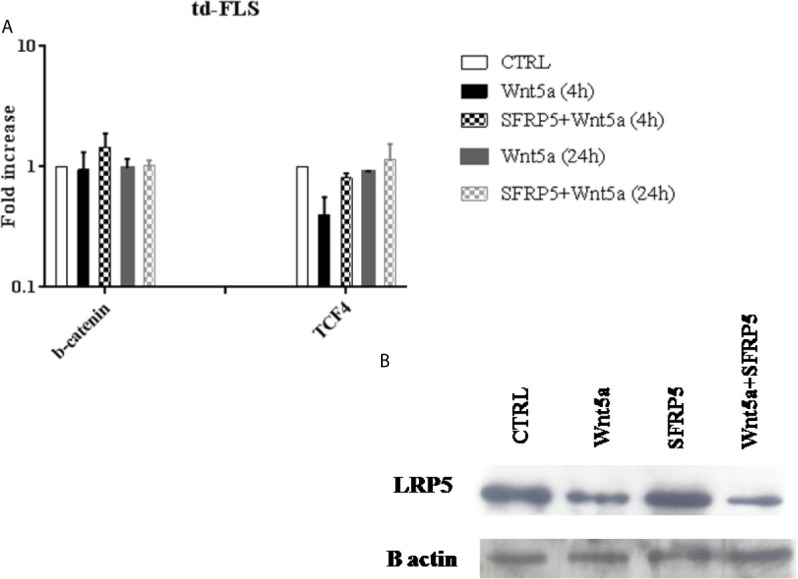
Effect of Wnt5a on the canonical Wnt pathway activation in RA td-FLS. RA td-FLS (passage 3) were stimulated with recombinant Wnt5a (300 ng/ ml) in the presence or absence of SFRP5 (1 mg/ml) for 4 h and/or 24h. Intracellular regulator expression profiles (β- catenin and TCF4) were determined in cell culture using qPCR **(A)**. Gene expression levels in unstimulated cells were assumed to be 1. Results are shown as the mean ± SD of two independent experiments each performed in triplicates using at least two different lots of each of the recombinant Wnt proteins. The protein abundance of LRP5 was studied by Western blot analysis after 24h of stimulation **(B)**. The Western blot is representative of one experiment out of two.

After Wnt5a stimulation for 4 h and 24 h, no changes in β- catenin expression were observed. The expression of the canonical transcription factor TCF4 decreased after 4h of Wnt5a stimulation and Wnt5a/SFRP5 treatment (**Figure 7A**). Consistent with this, td-FLS treatment with Wnt5a alone or associated with SFRP5 for 24h diminished the protein expression levels of the canonical coreceptor LRP5. It is worth noting that SFRP5 alone did not elicit LRP5 expression (**Figure 7B**).

## Discussion

RA is an acquired disorder that results from the concerted actions of different cell types both in the inflamed joint and in the circulation. RA td-FLS have a key role in all principal features of RA including hyperplasia and inflammation (3). Since neither increased proliferation nor inadequate apoptosis are totally responsible for the accumulation of RA td-FLS in the joint and synovial hyperplasia, there is certainly a cell migration phenomenon of progenitors from the blood to the synovium. The paper by Galligan and colleagues suggests that circulating fibrocytes are precursors of td-FLS and their activation in the circulation may be indicative of subsequent synovial inflammation and joint destruction (21). In a previous study, we showed that synovial fluid from RA patients gives rise to fd-FLS that contribute to the progression of RA (22). It is possible that fd-FLS derived from circulating fibrocytes (5).

As td-FLS are important in the pathogenesis of RA, the identification of endogenous molecules that regulate td-FLS function is of paramount importance. The evidence accumulated to date shows that the Wnt signaling pathway plays a critical role in the pathogenesis of RA. In fact, some members of the Wnt pathway, such as Wnt5a, have been identified in the synovial tissue and serum of patients with RA (9). Firstly, we found that Wnt5a expression was restricted to td-FLS from RA patients. Our results are consistent with Sen and colleague’s study showing that RA tissues express high gene levels of Wnt5a whereas OA tissues express either much less or almost no detectable Wnt5a mRNA (4). Subsequently, we demonstrated that Wnt5a was expressed in the two eventual precursors of td-FLS: fd-FlS and fibrocytes during RA.

Surprisingly, it has been possible to show greater expression of Wnt inhibitors such as SFRP in blood and synovial tissues during RA (23). Our data, demonstrated that SFRP5 was expressed in RA td-FLS, RA fd-FLS but not in RA fibrocytes. Indeed, as was shown by Kwon and colleagues, both RA td-FLS and PBMC express SFRP5 (24). SFRP can prevent Wnt interaction with some Fzd receptors and decrease Wnt signaling. However, in some contexts, SFRP proteins could facilitate Wnt signaling by enhancing Wnt diffusion (14, 23).

Recent reports identified Wnt5a to interact with various members of the Fzd receptor family (Fzd 4, 5, 8) as well as the receptors Ror2 and Ryk (9, 13, 25). Our data revealed that td-FLS, fd-FLS and fibrocytes expressed several Wnt5a receptors: Fzd4, Fzd5, Ror2, Ryk and the coreceptor LRP5 during RA. Interestingly, our study demonstrated that the expression profile of Fzd5 was particularly higher in fibrocytes compared to td-FLS and fd-FLS during RA. A previous report demonstrated that Fzd5 is expressed in RA tissues. Furthermore, Fzd5 levels are higher in RA synovium in comparison to osteoarthritis and normal adult synovium. This Wnt5a receptor plays an important role in FLS-mediated inflammatory response. RA td-FLS treatment with a polyclonal antibody specific for the extracellular domain of Fzd5 blocks IL6 and IL15 expression at both RNA and protein levels (9). Fzd5 plays a pivotal role not only in inflammation but also in cell migration (26). Considering all these results, Fzd5 may constitute one of the receptors involved in fibrocyte migration into the inflamed joint during RA.

Several research lines suggest that the Wnt5a-mediated signaling plays an important role in the induction of cytokines and other pro-inflammatory molecules in many cell types including neutrophils, monocytes, and endothelial cells (27–29). To understand the functional role of Wnt5a *in vitro*, RA td-FLS were stimulated with Wnt5a for 4 h and 24 h.Our data showed that Wnt5a enhanced rapidly the expression of IL1β, IL6, IL8, CCL2, CXCL10 and COX2 in RA td-FLS. According to Sen and colleague’s study, the transfection of RA td-FLS with either an antisense Wnt5a vector or a dnWnt5a down-regulates cytokine expression (9). Our data clearly demonstrated a robust induction of IL1β in response to Wnt5a in RA td-FLS. This cytokine plays a key role in invasiveness, inflammation and FLS activation (30). It may promote the formation of osteoclast‐like cells that can enlarge channels between the bone marrow and the synovial cavity (9). Wnt5a stimulated the expression of three important chemokines: IL8, CCL2 and CXCL10; it might stimulate leukocyte migration, predominantly neutrophils and fibrocytes (30).

Although the role of the inhibitor SFRP5 was characterized in some cell types, there is little to no evidence about its inflammatory function in RA td-FLS. Surprisingly, we found that RA td-FLS cytokine production induced by Wnt5a was not inhibited but enhanced in the presence of SFRP5. This observation is consistent with a previous report by Yu and colleagues who demonstrated that SFRP5 enhances the pro-inflammatory activity of Wnt5a in macrophages (31). In fact, IL12, IL6 and TNF levels were higher in macrophages treated with Wnt5a associated with SFRP5 than in macrophages stimulated by Wnt5a alone. A similar picture was seen when macrophages are treated with Wnt5a associated with the canonical inhibitors: SFRP1 or Dickkopf 1. These observations indicate that there is, in fact, a direct link between the pro-inflammatory effect of Wnt5a and the inhibition of the canonical Wnt pathway. In our study, we measured the gene expression of TCF4 and β-catenin after cell treatment. We were able to detect a decrease in the canonical transcription factor: TCF4 levels after Wnt5a addition alone or associated with SFRP5. However, we did not observe changes in β-catenin expression after cell stimulation. Our data agree with a previous report by Mikels and Nusse. The authors described that Wnt5a protein treatment has no effect on β-catenin levels but rather inhibits canonical Wnt signaling at the level of TCF transcription (25). Other researchers found that Wnt5a inhibits the canonical Wnt signaling by diminishing the gene expression of Axin2 which is an important element in the canonical cascade (31). In this study, we limited our exploration to an evaluation of the mRNA expression of β-catenin and we did not assess the phosphorylated form of this protein in RA td-FLS after Wnt5a and SFRP5 treatment. It is also believed that Wnt signaling pathways can act in an antagonistic manner. Various noncanonical Wnt signaling mechanisms have been reported to inhibit the β-catenin pathway by increasing β-catenin turnover or decreasing β-catenin/TCF association with DNA (32). It will also be interesting to explore the noncanonical Wnt pathways after cell treatment with Wnt5a and SFRP5.

Finally, we examined the effects of Wnt5a and SFRP5 on the canonical coreceptor LRP5. Our results indicated that the addition of Wnt5a alone and in conjunction with SFRP5 induced a clear drop in LRP5 protein expression. In fact, Wnt5a can activate the canonical and the noncanonical pathways depending on the cell type or the repertory of available receptors (13, 25, 33). A study conducted by Mikels and Nusse showed that Wnt5a activates the canonical Wnt pathway in the presence of both Fzd4 and LRP5. However, the interaction between Wnt5a and Ror2 inhibits the canonical Wnt pathway and activates a noncanonical Wnt signaling (25). The diversity of receptors that interact with Wnt and transduce their signals is matched by an equally diverse set of proteins that modulate Wnt activity in the extracellular space (34). Several molecules, including SFRP, act by forming nonfunctional complexes with some Wnt receptors and then reduce the possibilities of interaction between Wnt molecules and those receptors (35). As previously noted, noncanonical Wnt signaling can inhibit β-catenin transcriptional activity (32). Such a mechanism could be activated by some SFRP, such as SFRP5, *via* blocking particular receptors to enhance the interaction between Wnt and some noncanonical receptors (36). This could be the reason behind the enhanced pro-inflammatory response of Wnt5a when associated with SFRP5. The contradictory results between the studies that used recombinant SFRP5 (31) and the studies based on SFRP5 gene silencing (24) could perhaps be explained by the role of SFRP5 protein in controlling Wnt5a-Wnt receptor interactions.

Certain limitations should be considered in our study. The first limitation was related to the small number of RA patients and samples. While increasing the number of synovial fluid and blood samples was possible, it was difficult to obtain more fresh synovial tissue specimens for td-FLS cultures. Recent advances in managing patients with RA have resulted in reduced access to synovial tissues for researchers. A second limitation of our study was inherent to the exclusive use of cell cultures, which is known to be unable to recapitulate the full pathogenesis of RA. The third limitation was the absence of an evaluation of Wnt5a and SFRP5 effects on normal or OA td-FLS pro-inflammatory response. The findings may not be similar in non- RA cells, perhaps due to the unique aggressive characteristics of RA FLS and the cellular context. In addition, our study was limited to the evaluation of mRNA expression of the pro-inflammatory targets after Wnt5a and SFRP5 treatment. The evaluation of cytokine secretion in the supernatant remains to be established to firmly establish the importance of the Wnt5 pathway involvement in RA-FLS mediated inflammation.

## Conclusion

The findings of this study indicate that td-FLS and their eventual precursors: fd-FLS and fibrocytes express Wnt5a signaling elements during RA. The pro-inflammatory effects of Wnt5a on td-FLS are enhanced in the presence of SFRP5. Furthermore, the association of Wnt5a with SFRP5 stimulates RA td-FLS response through mechanisms involving the inhibition of TCF4 and LRP5. We provide Wnt5a signaling a new argument for a potential target of td-FLS-directed RA therapy.

## Data Availability Statement

The raw data supporting the conclusions of this article will be made available by the authors, without undue reservation.

## Ethics Statement

The studies involving human participants were reviewed and approved by The studies involving human participants were reviewed and approved by La Rabta Hospital ethic committee, Tunis, Tunisia. The patients/participants provided their written informed consent to participate in this study.

## Author Contributions

DM and WK: experiments and writing. NS, AM MM, LA, SJ, SR, and LT: experiments. MK-S, EC, and LL: concept and writing. All authors contributed to the article and approved the submitted version.

## Funding

The study was funded by grants from the Tunisian Ministry of High Education, Research and Technology.

## Conflict of Interest

The authors declare that the research was conducted in the absence of any commercial or financial relationships that could be construed as a potential conflict of interest.

## Abbreviations

RA, Rheumatoid Arthritis; FLS, fibroblast-like synoviocytes, SF, Synovial fluid; SFRP, Secreted Frizzled-Related Protein.

## References

[1] Nygaard G Firestein GS. Restoring Synovial Homeostasis in Rheumatoid Arthritis by Targeting Fibroblast-Like Synoviocytes. Nat Rev Rheumatol (2020) 16:316–33. 10.1038/s41584-020-0413-5 PMC7987137 32393826

[2] Vital EM Emery P. The Development of Targeted Therapies in Rheumatoid Arthritis. J Autoimmun (2008) 31:219–27. 10.1016/j.jaut.2008.04.006 18501558

[3] Filer A. The Fibroblast as a Therapeutic Target in Rheumatoid Arthritis. Curr Opin Pharmacol (2013) 13:413–19. 10.1016/j.coph.2013.02.006 23562164

[4] Sen M Lauterbach K El-Gabalawy H Firestein GS Corr M Carson DA. Expression and Function of Wingless and Frizzled Homologs in Rheumatoid Arthritis. Proc Natl Acad Sci USA (2000) 97:2791–96. 10.1073/pnas.050574297 PMC16008 10688908

[5] Stebulis JA Rossetti RG Atez FJ Zurier RB. Fibroblast-Like Synovial Cells Derived From Synovial Fluid. J Rheumatol (2005) 32:301–06. 15693092

[6] Machado CRL Resende GG Rafaela BV Macedo RBV do Nascimento CV Branco AS. Fibroblast-Like Synoviocytes From Fluid and Synovial Membrane From Primary Osteoarthritis Demonstrate Similar Production of Interleukin 6, and Metalloproteinases 1 and 3. Clin Exp Rheumatol (2018) 37:306–09. 30620271

[7] Zhao Y Wang CL Li RM Hui TQ Su YY Yuan Q. Wnt5a Promotes Inflammatory Responses *via* Nuclear Factor kappaB (NF-KappaB) and Mitogen-Activated Protein Kinase (MAPK) Pathways in Human Dental Pulp Cells. J Biol Chem (2014) 289:21028–39. 10.1074/jbc.A113.546523 PMC4110308 24891513

[8] Freese JL Pino D Pleasure SJ. Wnt Signaling in Development and Disease. Neurobiol Dis (2010) 38:148–53. 10.1016/j.nbd.2009.09.003 PMC2854277 19765659

[9] Sen M Chamorro M Reifert J Corr M Carson DA. Blockade of Wnt-5A/frizzled 5 Signaling Inhibits Rheumatoid Synoviocyte Activation. Arthritis Rheumatol (2001) 44:772–81. 10.1002/1529-0131(200104)44:4<772::AID-ANR133>3.0.CO;2-L 11315916

[10] Sato A Yamamoto H Sakane H Koyama H Kikuchi A. Wnt5a Regulates Distinct Signalling Pathways by Binding to Frizzled2. EMBO J (2010) 29:41–4. 10.1038/emboj.2009.322 PMC2808370 19910923

[11] Nusse R Clevers H. Wnt/Beta-Catenin Signaling, Disease, and Emerging Therapeutic Modalities. Cell (2017) 169:985–99. 10.1016/j.cell.2017.05.016 28575679

[12] Zarkou V Galaras A Giakountis A Hatzis P. Crosstalk Mechanisms Between the WNT Signaling Pathway and Long Non-Coding RNAs. Noncoding RNA Res (2018) 3:42–53. 10.1016/j.ncrna.2018.04.001 30159439 PMC6096407

[13] Rauner M Stein N Winzer M Goettsch C Zwerina J Schett G. WNT5A Is Induced by Inflammatory Mediators in Bone Marrow Stromal Cells and Regulates Cytokine and Chemokine Production. J Bone Miner Res (2012) 27:575–85. 10.1002/jbmr.1488 22162112

[14] Sen M. Wnt Signalling in Rheumatoid Arthritis. Rheumatol (Oxford) (2005) 44:708–13. 10.1093/rheumatology/keh553 15705634

[15] Ouchi N Higuchi A Ohashi K Oshima Y Gokce N Shibata R. Sfrp5 is an Anti-Inflammatory Adipokine That Modulates Metabolic Dysfunction in Obesity. Science (2010) 329:454–57. 10.1126/science.1188280 PMC3132938 20558665

[16] Nanbara H Wara-aswapati N Nagasawa T Yoshida Y Yashiro R Bando Y. Modulation of Wnt5a Expression by Periodontopathic Bacteria. PloS One (2012) 7:e34434. 10.1371/journal.pone.0034434 22485170 PMC3317782

[17] Zhan T Rindtorff N Boutros M. Wnt Signaling in Cancer. Oncogene (2017) 36:1461–73. 10.1038/onc.2016.304 PMC5357762 27617575

[18] Aletaha D Neogi T Silman AJ Funovits J Felson DT Bingham CO. 2010 Rheumatoid Arthritis Classification Criteria: An American College of Rheumatology/European League Against Rheumatism Collaborative Initiative. Ann Rheum Dis (2010) 69:1580–88. 10.1136/ard.2010.138461 20699241

[19] Pilling D Tucker NM Gomer RH. Aggregated IgG Inhibits the Differentiation of Human Fibrocytes. J Leukoc Biol (2006) 79:1242–51. 10.1189/jlb.0805456 PMC4482138 16543402

[20] Pilling D Cox N Vakil V Verbeek JS Gomer RH. The Long Pentraxin PTX3 Promotes Fibrocyte Differentiation. PloS One (2015) 10:e0119709. 10.1371/journal.pone.0119709 25774777 PMC4361553

[21] Galligan CL Keystone EC Fish EN. Fibrocyte and T Cell Interactions Promote Disease Pathogenesis in Rheumatoid Arthritis. J Autoimmun (2016) 69:38–50. 10.1016/j.jaut.2016.02.008 26948996

[22] Elhaj Mahmoud D Sassi N Drissi G Barsaoui M Zitouna K Sahli H. sFRP3 and DKK1 Regulate Fibroblast-Like Synoviocytes Markers and Wnt Elements Expression Depending on Cellular Context. Immunol Invest (2017) 46:314–28. 10.1080/08820139.2016.1267204 28151034

[23] Mii Y Taira M. Secreted Frizzled-related Proteins Enhance the Diffusion of Wnt Ligands and Expand Their Signalling Range. Development (2009) 136:4083–88. 10.1242/dev.032524 19906850

[24] Kwon YJ Lee SW Park YB Lee SK Park MC. Secreted Frizzled-Related Protein 5 Suppresses Inflammatory Response in Rheumatoid Arthritis Fibroblast-Like Synoviocytes Through Down-Regulation of C-Jun N-Terminal Kinase. Rheumatology (2014) 53:1704–11. 10.1093/rheumatology/keu167 24764263

[25] Mikels AJ Nusse R. Purified Wnt5a Protein Activates or Inhibits Beta-Catenin-TCF Signaling Depending on Receptor Context. PloS Biol (2006) 4:e115. 10.1371/journal.pbio.0040115 16602827 PMC1420652

[26] Brandt MM van Dijk CGM Chrifi I Kool HM Burgisser PE Louzao-Martinez L. Endothelial Loss of Fzd5 Stimulates PKC/Ets1-Mediated Transcription of Angpt2 and Flt1. Angiogenesis (2018) 21:805–21. 10.1007/s10456-018-9625-6 PMC6208898 29845518

[27] Jung YS Lee HY Kim SD Park JS Kim JK Suh PG. Wnt5a Stimulates Chemotactic Migration and Chemokine Production in Human Neutrophils. Exp Mol Med (2013) 45:e27. 10.1038/emm.2013.48 23764954 PMC3701286

[28] Kim J Chang W Jung Y Song K Lee I. Wnt5a Activates THP-1 Monocytic Cells *via* a Beta-Catenin-Independent Pathway Involving JNK and NF-KappaB Activation. Cytokine (2012) 60:242–48. 10.1016/j.cyto.2012.06.013 22763043

[29] Kim J Kim J Kim DW Ha Y Ihm MH Kim H. Wnt5a Induces Endothelial Inflammation *via* Beta-Catenin-Independent Signaling. J Immunol (2010) 185:1274–82. 10.4049/jimmunol.1000181 20554957

[30] Suzuki M Tetsuka T Yoshida S Watanabe N Kobayashi M Matsui N. The Role of p38 Mitogen-Activated Protein Kinase in IL-6 and IL-8 Production From the TNF-Alpha- or IL-1Beta-Stimulated Rheumatoid Synovial Fibroblasts. FEBS Lett (2000) 465:23–7. 10.1016/s0014-5793(99)01717-2 10620700

[31] Yu CH Nguyen TT Irvine KM Sweet MJ Frazer IH Blumenthal A. Recombinant Wnt3a and Wnt5a Elicit Macrophage Cytokine Production and Tolerization to Microbial Stimulation *via* Toll-like Receptor 4. Eur J Immunol (2014) 44:1480–90. 10.1002/eji.201343959 24643512

[32] Topol L Jiang X Choi H Garrett-Beal L Carolan PJ Yang Y. Wnt-5a Inhibits the Canonical Wnt Pathway by Promoting GSK-3-Independent Beta-Catenin Degradation. J Cell Biol (2003) 162:899–908. 10.1083/jcb.200303158 12952940 PMC2172823

[33] Boucher P Matz RL Terrand J. Atherosclerosis: Gone With the Wnt? Atherosclerosis (2020) 301:15–22. 10.1016/j.atherosclerosis.2020.03.024 32289618

[34] Grainger S Willert K. Mechanisms of Wnt Signaling and Control. Wiley Interdiscip Rev Syst Biol Med (2018) 10:e1422. 10.1002/wsbm.1422 PMC6165711 29600540

[35] Cruciat CM Niehrs C. Secreted and Transmembrane Wnt Inhibitors and Activators. Cold Spring Harb Perspect Biol (2013) 5:a015081. 10.1101/cshperspect.a015081 23085770 PMC3578365

[36] Xavier CP Melikova M Chuman Y Üren A Baljinnyam B Rubin JS. Secreted Frizzled-Related Protein Potentiation Versus Inhibition of Wnt3a/β-Catenin Signaling. Cell Signal (2014) 26:94–101. 10.1016/j.cellsig.2013.09.016 24080158 PMC3953133

